# Uncommon manifestation of Ebstein anomaly: A case report of apical displacement involving all tricuspid valve leaflets

**DOI:** 10.1002/ccr3.8991

**Published:** 2024-05-24

**Authors:** Amirhossein Jalali, Zahra Khajali, Mozhgan Parsaee, Soudabe Behrooj, Pegah Salehi, Mahsa Akbarian, Sara Shemshadi, Dariush Hooshyar

**Affiliations:** ^1^ Department of Cardiovascular Surgery, Division of Congenital Cardiac Surgery, Rajaie Cardiovascular Medical and Research Institute, Iran University of Medical Science Tehran Iran; ^2^ Echocardiography Research Center, Rajaie Cardiovascular Medical and Research Center Iran University of Medical Sciences Tehran Iran; ^3^ Student Research Committee, Faculty of Medicine Hormozgan University of Medical Sciences Bandar Abbas Iran

**Keywords:** annuloplasty, apical displacement, congenital heart disease, Ebstein anomaly, tricuspid valve displacement, unique surgical outcomes

## Abstract

**Key Clinical Message:**

This case report presents an uncommon variant of Ebstein anomaly, where all three tricuspid valve leaflets exhibited apical displacement—a rare finding. It illustrates the complexities in diagnosing and managing such atypical presentations, with successful surgical correction through annuloplasty. The report adds valuable insights to the limited literature on this congenital heart disease.

**Abstract:**

Ebstein anomaly (EA), a rare congenital heart disorder, presents with diverse clinical spectrums. This case report explores a distinctive manifestation of EA, where all three tricuspid valve (TV) leaflets exhibited apical displacement, highlighting a novel aspect in the presentation of this condition. A 44‐year‐old woman, under long‐term medical surveillance for EA, showcased an atypical clinical trajectory marked by the apical displacement of all TV leaflets, which is uncommon in EA. Despite a predominantly asymptomatic course, recent exacerbation of symptoms prompted further evaluation. Diagnostic modalities, including echocardiography and cardiac magnetic resonance imaging, revealed severe tricuspid regurgitation concomitant with unprecedented apical displacement of the anterior, septal, and posterior tricuspid leaflets. The displacement of the anterior leaflet was contrary to typical embryonic valvular formation expectations, indicating a unique presentation within EA. The patient underwent annuloplasty surgery, which successfully rectified the anomalous TV architecture. Postoperative evaluation demonstrated mild residual tricuspid regurgitation, and the patient was discharged in stable condition. This case underscores the variability in EA presentations and accentuates the significance of tailored surgical interventions. The observation of apical displacement involving all TV leaflets adds a unique dimension to the existing EA literature, reinforcing the need for careful diagnosis and personalized treatment approaches.

## 1 INTRODUCTION

Ebstein anomaly (EA), a rare congenital heart disease identified in 1866, occurs in about 1 in 200,000 live births.^1^, ^2^ This condition, present from birth, displays varied symptoms across life stages, dependent on disease severity.^3^ In a normal heart, the tricuspid valve (TV) has three leaflets (anterior, posterior, and septal).^2^ In typical cases of EA, the septal and posterior leaflets are apically displaced due to a failure of delamination during embryologic development. The anterior leaflet generally maintains its normal position or exhibits less severe displacement. A key feature is the downward displacement of the posterior and septal TV leaflets.^1^, ^4^, ^5^ This case report stands out due to the apical displacement of all three leaflets of the TV, a highly unusual presentation in EA. This unique displacement pattern suggests significant implications for diagnosis and treatment, providing a new perspective on the variability of EA presentations.

## 2 CASE HISTORY

A 44‐year‐old woman with no family history of cardiovascular diseases had been under medical supervision and treatment since birth, diagnosed with Ebstein's anomaly. The patient had been asymptomatic and free from clinical complaints throughout her life, following a medication regimen that included allopurinol (100 mg daily), metoprolol succinate (47.5 mg twice daily), captopril (25 mg twice daily), rivaroxaban (20 mg daily), and furosemide (20 mg three times a day).

During her previous visit to this medical center in October 2019, the patient had stable vital signs, and cardiac magnetic resonance imaging (Cardiac MRI) indicated a normal cardiothoracic ratio (CTR). However, at her recent visit, she reported heart palpitations and increasing shortness of breath, both during exertion and at rest, which worsened on the day of admission to Shahid Rajaei Hospital.

On initial examination, the electrocardiogram (EKG) revealed atrial fibrillation with rapid ventricular response (AFib with RVR). The patient received digoxin (0.5 mg/2 mL injection) for rate control, resulting in stabilization of her vital signs, with Blood Pressure (BP): 90/50 mmHg, Pulse Rate (PR): 74 bpm, and Oxygen Saturation (SPO_2_): 96%. Despite stabilization, the patient continued to experience shortness of breath with decreased activity tolerance.

## 3 METHODS

**FIGURE 1:**
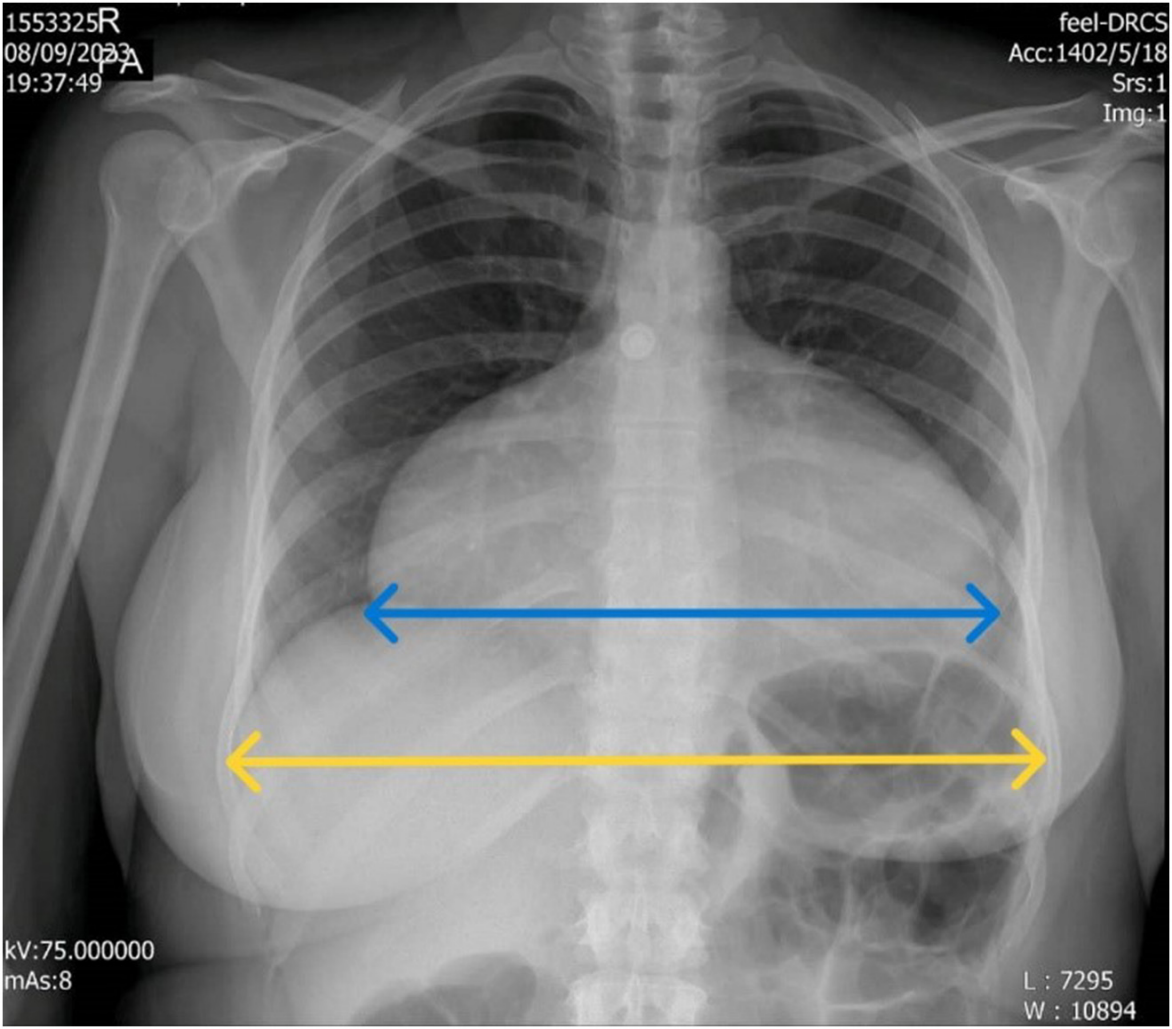
Increased Cardiothoracic Ratio in patient posterior–anterior chest X‐ray.

**FIGURE 2:**
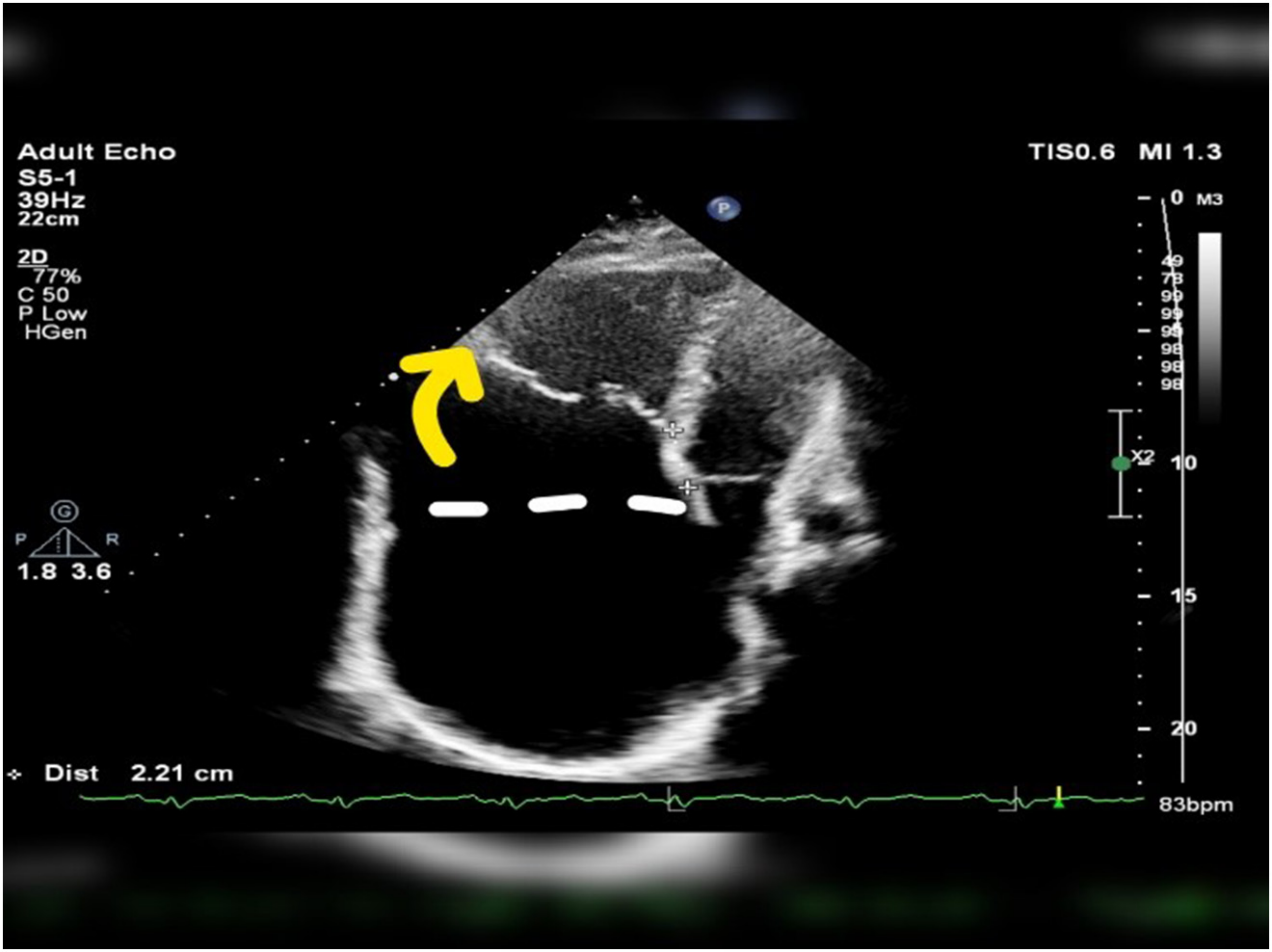
Apical 4chamber view showing displacement of anterior tricuspid leaflet (5.3 cm, yellow arrow) relative to tricuspid annulus in addition to displacement of septal leaflet (2.21 cm).

A posterior–anterior chest X‐ray showed an elevated cardiothoracic ratio (>65%) (Figure 1). Echocardiography revealed anterior tricuspid leaflet displacement, along with septal and posterior leaflet displacement. The echocardiogram also identified severe tricuspid regurgitation. Transesophageal echocardiography (TEE) showed a small‐sized left ventricular (LV) cavity with mild systolic dysfunction (EF: 50%), without LV hypertrophy. The right ventricle (RV) demonstrated significant atrialization, with severe enlargement and moderate functional RV dysfunction (Figure 2; Video S1).

The left atrium (LA) was small, while the right atrium (RA) had severe enlargement due to atrialization of the RV. No clot or smoke was observed in the LA and the left atrial appendage, with normal drainage of all pulmonary veins. The mitral valve exhibited mild prolapse, without significant mitral stenosis, and only trivial mitral regurgitation. There was no pulmonary arterial hypertension, and the pulmonary valve was normal with mild to moderate pulmonary insufficiency. The inferior vena cava was of normal size and exhibited respiratory collapse without evidence of pulmonary embolism.

**FIGURE 3:**
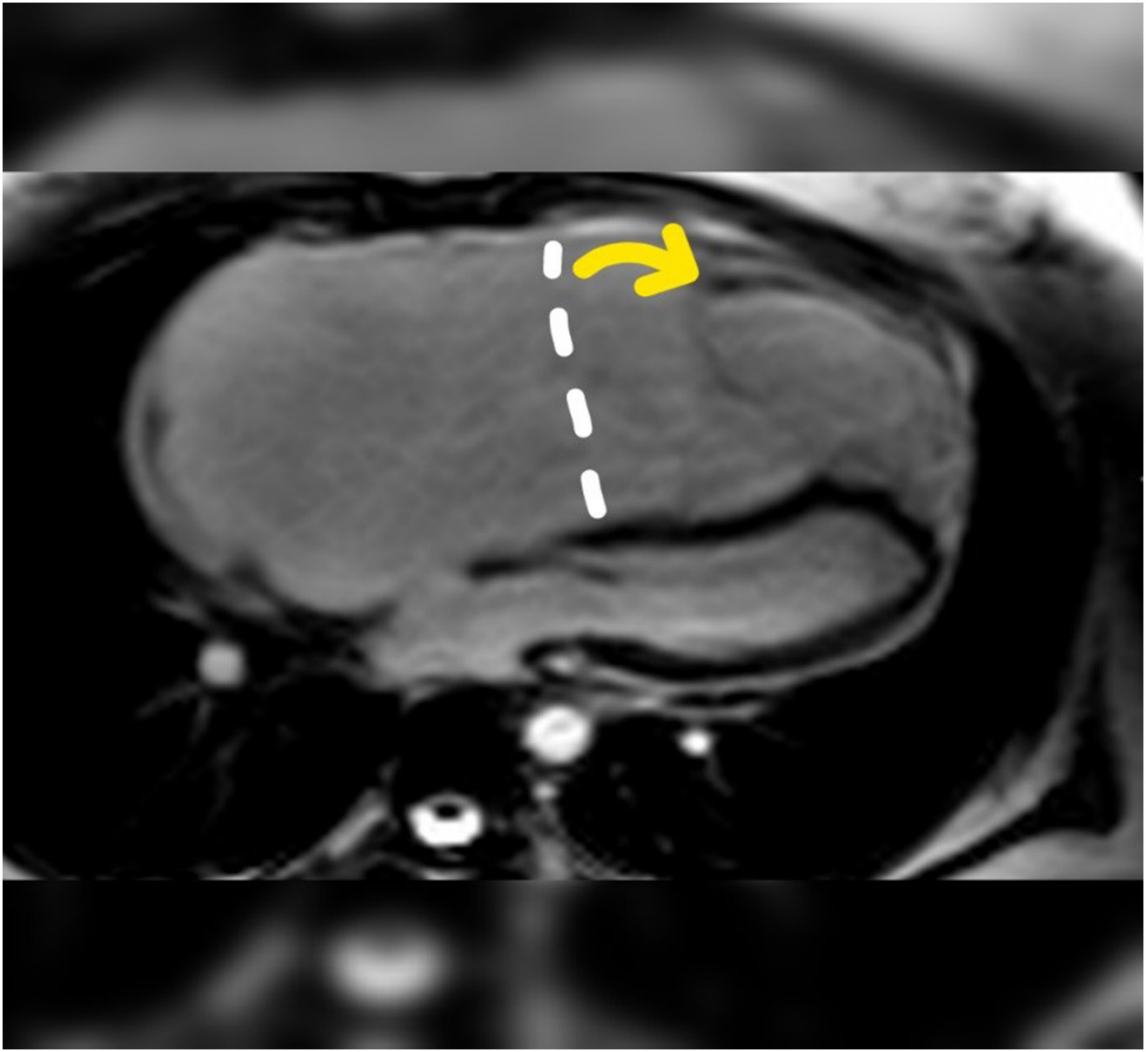
Apical displacement of tricuspid anterior leaflet (5.3 cm, yellow arrow) relative to tricuspid annulus in cardiac magnetic resonance imaging.

Cardiac MRI confirmed anterior leaflet displacement, consistent with echocardiography findings (Figure 3). Given these diagnostic results and the patient's symptoms, annuloplasty surgery was recommended.

## 4 SURGICAL PROCEDURE

Under general anesthesia, the patient underwent cardiothoracic surgical procedures involving median sternotomy and cardiopulmonary bypass (CPB), with moderate to deep hypothermia. The operation included surgical dissection to reduce the width of the atrialized portion of the right atrium, with TV repair using horizontal plication of the RV with a 38 mm prosthetic ring (Medtronic, CG future annuloplasty ring, Model 638BL38). Additional procedures involved papillary muscle approximation, edge‐to‐edge repair, and plication of the posterior leaflet's free edge.

Following the saline test and confirmation of tricuspid regurgitation resolution, the surgery proceeded with the closure of the patent foramen ovale (PFO), aortic declamping, and successful separation from CPB (Video S2). Transesophageal echocardiography confirmed normal ventricular function and elimination of tricuspid regurgitation.

## 5 RESULTS

The surgical procedures concluded successfully, with no complications. The patient regained sinus rhythm and was weaned from CPB without difficulty. Following careful hemostasis, she was transferred to the Cardiovascular Surgery Intensive Care Unit (ICU). The patient was eventually discharged in stable condition with prescriptions for beta‐blockers, anticoagulants, and furosemide. Follow‐up appointments were scheduled to monitor her recovery and evaluate long‐term outcomes.

## 6 DISCUSSION

Ebstein's anomaly represents a distinct facet of TV involvement in congenital heart diseases, characterized by the downward displacement of the septal leaflet and atrialized RV. This anomaly manifests as a structural malformation involving the TV and RV, marked by the adherence of the septal and posterior leaflets to the underlying myocardium—a consequence of delamination failure during embryonic development. The functional annulus experiences a pronounced downward shift, defined as the descent of the posterior and septal TV leaflets by more than 8 mm/m^2^ of body surface area from the anterior mitral valve leaflet. Simultaneously, the “atrialized” portion of the RV undergoes dilation, exhibiting variable degrees of hypertrophy and wall thinning. Salient features include redundancy, fenestrations, and tethering of the anterior leaflet, coupled with dilation of the right atrioventricular junction, referred to as the true tricuspid annulus.^1^, ^4^, ^6^

In contrast to the septal and posterior leaflets, the anterior leaflet typically maintains its normative position due to its distinct embryonic origin from the lateral endocardial cushion and lateral conus. Consequently, Ebstein's anomaly rarely affects the anterior tricuspid leaflet, and the attachment point of the septal and inferior leaflets seldom experiences apical displacement beyond the junction between the ventricular inlet and the apicotrabecular component of the RV. Furthermore, the junctional hinge of the anterior leaflet is infrequently affected.^7^, ^8^ The anterior leaflet may exhibit variations, presenting as either large and sail‐like or tethered by fibrous tissue, resulting in reduced mobility.^9^ Mobility is intricately linked to chordal attachment, with very mobile anterior leaflets often lacking appropriate attachment, while tethered leaflets may directly insert into a papillary muscle or the myocardium.^10^ The presence of fenestrations in the anterior leaflet significantly contributes to tricuspid regurgitation.^5^

A distinctive aspect observed in our study pertains to the downward (apical) displacement of the anterior TV leaflet, alongside the septal and posterior leaflets, contrary to anticipated outcomes based on embryonic valvular formation. Additionally, an observable cleft in the anterior leaflet lacks tethering. Tricuspid regurgitation, a common outcome in Ebstein's anomaly attributed to the ineffective coaptation of abnormal tricuspid leaflets, is also evident in our study's patient.^9^ Concurrently, prevalent congenital anomalies such as atrial septal defect and PFO, occurring in over 80% of patients, align with the findings in our study.^11^

While Ebstein's anomaly is typically diagnosed prenatally or in early childhood, some patients receive a new diagnosis in adulthood, presenting with symptoms encompassing dyspnea, fatigue, and lower extremity edema.^9^ Exercise intolerance may ensue from diminished right ventricular function, severe tricuspid regurgitation, or exercise‐induced cyanosis arising from right‐to‐left shunting across the atrial septum.

The management approach for asymptomatic patients primarily involves medical observation, whereas symptomatic individuals or those exhibiting worsened exercise capacity, cyanosis, paradoxical embolism, progressive RV dilation or dysfunction, or the onset of arrhythmias warrant consideration for operative intervention. The focal point of operative intervention is TV repair, often entailing RV plication, right atrial reduction, and atrial septal closure or subtotal closure. Notably, tricuspid replacement in adult patients has demonstrated safety and efficacy.^5^

Existing literature has reported instances of isolated anterior TV leaflet apical displacement.^12^, ^13^, ^14^ However, our investigation reveals that only one other documented case involves apical displacement alongside other leaflets.^8^

Given the diverse spectrum of manifestations, ranging from asymptomatic to symptomatic necessitating drug or surgical intervention, a nuanced understanding of the disease's myriad expressions and the associated anatomical and congenital changes in the heart is imperative. This comprehension facilitates informed decisions regarding drug treatments and aids in mitigating complications. Additionally, the recognition of the various anatomical variations in Ebstein's anomaly in relation to diseases can empower surgeons to make well‐informed decisions concerning surgical interventions and techniques.

## 7 CONCLUSION

The unique combination of apical displacement of the anterior TV leaflet with additional structural anomalies makes this case exceptional within the context of Ebstein's anomaly. It underscores the need for meticulous cardiac assessment and personalized surgical strategies, emphasizing the complex nature of congenital heart diseases. As demonstrated in this case, achieving successful surgical outcomes requires comprehensive understanding and tailored interventions, informed by detailed anatomical insights.

## AUTHOR CONTRIBUTIONS

**Amirhossein Jalali:** Data curation; supervision. **Zahra Khajali:** Data curation; supervision. **Mozhgan Parsaee:** Data curation; supervision. **Soudabe Behrooj:** Data curation; writing – review and editing. **Pegah Salehi:** Data curation. **Mahsa Akbarian:** Data curation. **Sara Shemshadi:** Data curation. **Dariush Hooshyar:** Data curation; resources; writing – original draft; writing – review and editing.

## FUNDING INFORMATION

This study did not receive financial support. The costs associated with this study were covered by the authors.

## CONFLICT OF INTEREST STATEMENT

The authors declare no conflicts of interest.

## ETHICS STATEMENT

In compliance with the patient journal's consent policy, written informed consent was acquired from the patient for the publication of this report. The authors obtained a waiver for ethical approval from the institution review board committee.

## CONSENT

Written informed consent was obtained from the patient for the publication of this report, in accordance with the patient journal's consent policy.

## Supporting information


Video S1.

Video S2.

## Data Availability

Data openly available in a public repository that issues datasets with DOIs.
